# Experimental Lymphedema: Can Cellular Therapies Augment the Therapeutic Potential for Lymphangiogenesis?

**DOI:** 10.1161/JAHA.112.003400

**Published:** 2012-08-24

**Authors:** Stanley G. Rockson

**Affiliations:** Stanford Center for Lymphatic and Venous Disorders, Division of Cardiovascular Medicine, Stanford University School of Medicine, Stanford, CA

**Keywords:** editorials, lymphangiogenesis, lymphedema

The lymphatic system, the subject of centuries of paradoxical relative neglect, is finally and deservedly coming into focus.^1–2^ This integral component of the mammalian vasculature plays a central role in immunocompetence and fluid homeostasis and, therefore, is often an active participant in the progression of disease.^3–4^

The biology of regional lymphatic vascular insufficiency is complex. When regional lymphatic flow is insufficient to maintain tissue fluid homeostasis, interstitial fluid accumulates and swelling ensues. Furthermore, in addition to this readily observed role in the maintenance of tissue fluid homeostasis, functioning lymphatics are crucial to the traffic of immunocompetent cells from the tissue periphery to the lymph node, where antigenic processing can occur.^5^

Lymphedema is the all-too-frequent clinical consequence of impaired lymphatic function. The condition does not typically threaten survival, yet the advent of lymphedema can significantly undermine productivity and quality of life for affected individuals. The sequelae of lymphedema include loss of function, restriction of movement, risk of infection, and profound alterations in psychosocial adjustment^6^ that include fear, affective disorders, and loss of self-esteem and body image.^7^ Lymphedema is a chronic debilitating disease for which there continues to be a great deal of clinical confusion and treatment delay.^8^

The current available treatments for lymphedema do not address the causal molecular pathophysiology and thus provide only modest delays in the progression to end-stage sequelae of lymphedema.^9^ In recent years, however, the identification of the molecular components of the lymphatic developmental apparatus has made it feasible to contemplate the therapeutic administration of lymphatic-specific growth factors (therapeutic lymphangiogenesis) to alleviate the tissue consequences of lymphatic vascular insufficiency.^10–15^

Despite initial perceptions of benefit, the enthusiasm for growth factor therapy has not been universal. The precise mechanism for vascular endothelial growth factor receptor 3–mediated amelioration of secondary lymphangiogenesis is unclear. There are currently contrasting observations that exogenous growth factor administration to sites of lymphatic injury augments early lymphatic endothelial cell proliferation but without the development of functionally competent vasculature.^16–18^ Furthermore, it seems that the ultimate ability of the lymphatic vasculature to regenerate after injury might be governed by an exquisite balance between prolymphangiogenic and antilymphangiogenic cytokines.^19^

In the current issue of the *Journal of the American Heart Association*, Shimizu et al^20^ address this persistent quandary in experimental therapeutics with a modification of the prolymphangiogenic approach. Specifically, they administered adipose-derived regenerative cells to a murine tail model of acquired lymphedema to assess whether these cells might serve to augment meaningful lymphatic neovascularization. The findings of this investigation include the observation of a direct lymphangiogenic response to implantation of adipose-derived regenerative cells into the zone of lymphovascular deficiency. In addition, the researchers observe that these cells in culture produce vascular endothelial growth factor C, which is capable of augmenting lymphangiogenesis. Finally, they report that the in vivo administration of the cellular material resulted in augmented recruitment of bone marrow–derived M2 macrophages that could serve the role of lymphatic endothelial progenitor cells. These findings are certainly congruent with other recent observations.^21^

The functional significance of the authors' findings ultimately must be demonstrated through further investigation. First, it must be acknowledged that the animal models for lymphedema are imperfect.^22^ The authors used a frequently studied small rodent model of acquired lymphedema^11–13,16–19^ that offers a high-throughput means to readily observe histological, microvascular, and molecular events. However, the ability to translate therapeutic observations in this model to human pathology might be limited. Cytokine-stimulated therapeutic lymphangiogenesis, whether via direct ligand administration or, as used here, indirect cell-mediated augmentation, simply might be insufficient to foster the development of the competent lymphatic architecture that is needed to restore lymph flow. Nevertheless, continued investigation and the creative approaches described here certainly are warranted. Patients with lymphedema undoubtedly will one day be the beneficiaries of these focused investigations.

## References

[1] Lee B Bergan J Rockson S Lymphedema: A Concise Compendium of Theory and Practice. 2011 1st London Springer

[2] Choi I Lee S Hong YK The new era of the lymphatic system: no longer secondary to the blood vascular system. Cold Spring Harb Perspect Med. 2012;2:a006445 22474611 10.1101/cshperspect.a006445 PMC3312397

[3] Oliver G Lymphatic vasculature development. Nat Rev Immunol. 2004;4:35-45 14704766 10.1038/nri1258

[4] Norrmen C Tammela T Petrova TV Alitalo K Biological basis of therapeutic lymphangiogenesis. Circulation. 2011;123:1335-1351 21444892 10.1161/CIRCULATIONAHA.107.704098

[5] Hodge LM Downey HF Lymphatic pump treatment enhances the lymphatic and immune systems. Exp Biol Med (Maywood). 2011;236:1109-1115 21865405 10.1258/ebm.2011.011057

[6] Velanovich V Szymanski W Quality of life of breast cancer patients with lymphedema. Am J Surg. 1999;177:184-187 discussion 188 10219851 10.1016/s0002-9610(99)00008-2

[7] Ridner SH The psycho-social impact of lymphedema. Lymphat Res Biol. 2009;7:109-112 19534633 10.1089/lrb.2009.0004 PMC2904185

[8] Szuba A Shin WS Strauss HW Rockson S The third circulation: radionuclide lymphoscintigraphy in the evaluation of lymphedema. J Nucl Med. 2003;44:43-57 12515876

[9] Nakamura K Rockson SG Molecular targets for therapeutic lymphangiogenesis in lymphatic dysfunction and disease. Lymphat Res Biol. 2008;6:181-189 19093791 10.1089/lrb.2008.63404

[10] Szuba A Skobe M Karkkainen MJ Shin WS Beynet DP Rockson NB Dakhil N Spilman S Goris ML Strauss HW Quertermous T Alitalo K Rockson SG Therapeutic lymphangiogenesis with human recombinant VEGF-C. FASEB J. 2002;16:U114-U130 10.1096/fj.02-0401fje 12397087

[11] Cheung L Han J Beilhack A Joshi S Wilburn P Dua A An A Rockson SG An experimental model for the study of lymphedema and its response to therapeutic lymphangiogenesis. BioDrugs. 2006;20:363-370 17176124 10.2165/00063030-200620060-00007

[12] Yoon YS Murayama T Gravereaux E Tkebuchava T Silver M Curry C Wecker A Kirchmair R Hu CS Kearney M Ashare A Jackson DG Kubo H Isner JM Losordo DW VEGF-C gene therapy augments postnatal lymphangiogenesis and ameliorates secondary lymphedema. J Clin Invest. 2003;111:717-725 12618526 10.1172/JCI15830 PMC151891

[13] Saito Y Nakagami H Morishita R Takami Y Kikuchi Y Hayashi H Nishikawa T Tamai K Azuma N Sasajima T Kaneda Y Transfection of human hepatocyte growth factor gene ameliorates secondary lymphedema via promotion of lymphangiogenesis. Circulation. 2006;114:1177-1184 16952986 10.1161/CIRCULATIONAHA.105.602953

[14] Jin da P An A Liu J Nakamura K Rockson SG Therapeutic responses to exogenous VEGF-C administration in experimental lymphedema: immunohistochemical and molecular characterization. Lymphat Res Biol. 2009;7:47-57 19302023 10.1089/lrb.2009.0002

[15] Choi I Lee S Kyoung Chung H Suk Lee Y Eui Kim K Choi D Park EK Yang D Ecoiffier T Monahan J Chen W Aguilar B Lee HN Yoo J Koh CJ Chen L Wong AK Hong YK 9-Cis retinoic acid promotes lymphangiogenesis and enhances lymphatic vessel regeneration: therapeutic implications of 9-cis retinoic acid for secondary lymphedema. Circulation. 2012;125:872-882 22275501 10.1161/CIRCULATIONAHA.111.030296 PMC3327127

[16] Goldman J Le TX Skobe M Swartz MA Overexpression of VEGF-C causes transient lymphatic hyperplasia but not increased lymphangiogenesis in regenerating skin. Circ Res. 2005;96:1193-1199 15890974 10.1161/01.RES.0000168918.27576.78

[17] Goldman J Conley KA Raehl A Bondy DM Pytowski B Swartz MA Rutkowski JM Jaroch DB Ongstad EL Regulation of lymphatic capillary regeneration by interstitial flow in skin. Am J Physiol Heart Circ Physiol. 2007;292:H2176-H2183 17189348 10.1152/ajpheart.01011.2006

[18] Rutkowski JM Moya M Johannes J Goldman J Swartz MA Secondary lymphedema in the mouse tail: lymphatic hyperplasia, VEGF-C upregulation, and the protective role of MMP-9. Microvasc Res. 2006;72:161-171 16876204 10.1016/j.mvr.2006.05.009 PMC2676671

[19] Zampell JC Avraham T Yoder N Fort N Yan A Weitman ES Mehrara BJ Lymphatic function is regulated by a coordinated expression of lymphangiogenic and anti-lymphangiogenic cytokines. Am J Physiol Cell Physiol. 2012;302:C392-C404 21940662 10.1152/ajpcell.00306.2011 PMC3328842

[20] Shimizu Y Shibata R Shintani S Ishii M Murohara T Therapeutic lymphangiogenesis with implantation of adipose-derived regenerative cells. J Am Heart Assoc. 2012;1:e000877 doi: 10.1161/JAHA.112.000877 10.1161/JAHA.112.000877 PMC3487362 23130156

[21] Yan A Avraham T Zampell JC Haviv YS Weitman E Mehrara BJ Adipose-derived stem cells promote lymphangiogenesis in response to VEGF-C stimulation or TGF-β1 inhibition. Future Oncol. 2011;7:1457-1473 22112321 10.2217/fon.11.121 PMC3263831

[22] Shin WS Rockson SG Animal models for the molecular and mechanistic study of lymphatic biology and disease. Ann N Y Acad Sci. 2008;1131:50-74 18519959 10.1196/annals.1413.005

